# Supplementary material for: Short-Term ONX-0914 Administration: Performance and Muscle Phenotype in Mdx Mice
Source: Int J Environ Res Public Health. 2020 Jul 19;17(14):5211. doi: 10.3390/ijerph17145211 (PMC7399807; doi:10.3390/ijerph17145211)
Supplement: Supplementary file 1 [file ijerph-17-05211-s001.pdf]

Short-term ONX-0914 Administration: Performance and muscle phenotype in *Mdx* mice

Dongmin. Kwak[1], Guoxian. Wei[1], LaDora V. Thompson[1]*, Jong-Hee Kim[2]*

[1]Department of Physical Therapy and Athletic Training, Boston University, Boston, MA.
[2]Department of Physical Education, Hanyang University, Seoul, South Korea.

*Equal contribution and corresponding authors

Correspondence:
[1] LaDora V. Thompson
Boston University
College of Health & Rehabilitation Sciences: Sargent College
Department of Physical Therapy & Athletic Training
635 Commonwealth Ave., Boston, MA 02215

[2] Jong-Hee Kim
Hanyang University
College of Performing Arts and Sport
Department of Physical Education
222 Wangsimni-ro, Seongdong-gu, Seoul 04763

ONLINE SUPPLEMENTARY MATERIAL

Supplemental Figure 1.

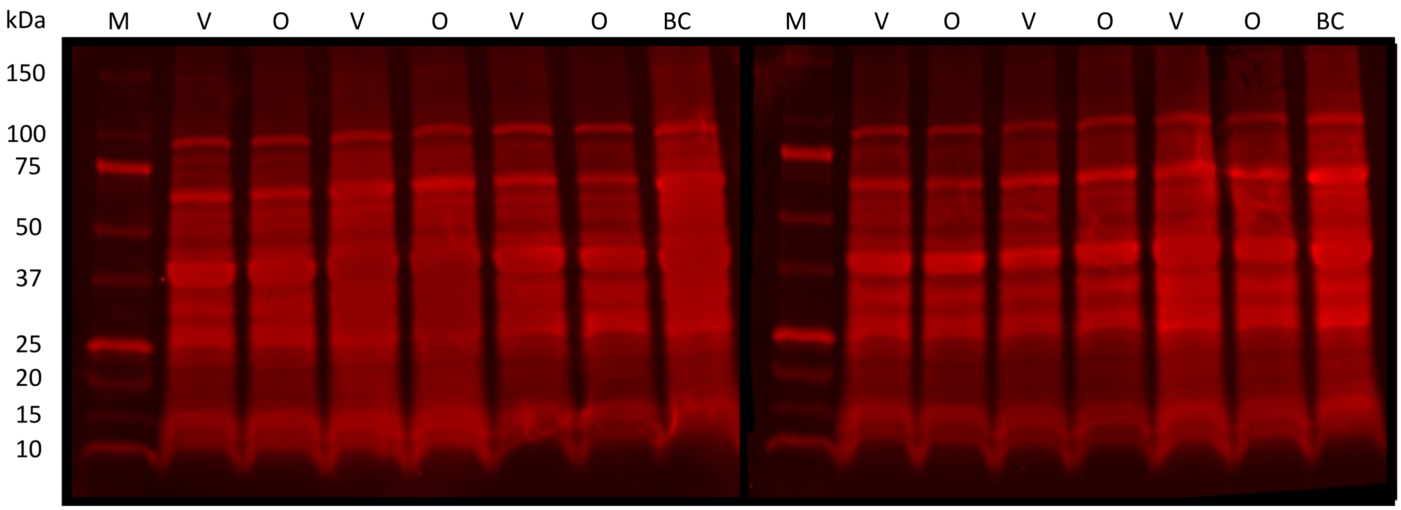

Representative image of total proteins from the gastrocnemius muscles (enriched proteasome preparation). The blot was stained with REVERT™ Total Protein Stain (licor.com/revert) by following REVERT™ Total Protein Stain Normalization Protocol, https://www.licor.com/documents/1q6nvqiov23om7n80hxo046w9mpfqx3s).The stained blots were imaged at 700 nm channel (red) with Odyssey imaging system (Li-Cor Biosciences, Lincoln, NE). M: Standard protein markers (kDa), BC: blot control (20 µg from one gastrocnemius in WT-V group), ONX-0914 treated MDX mouse (O) and vehicle treated MDX mouse (V).

Supplemental Figure 2.

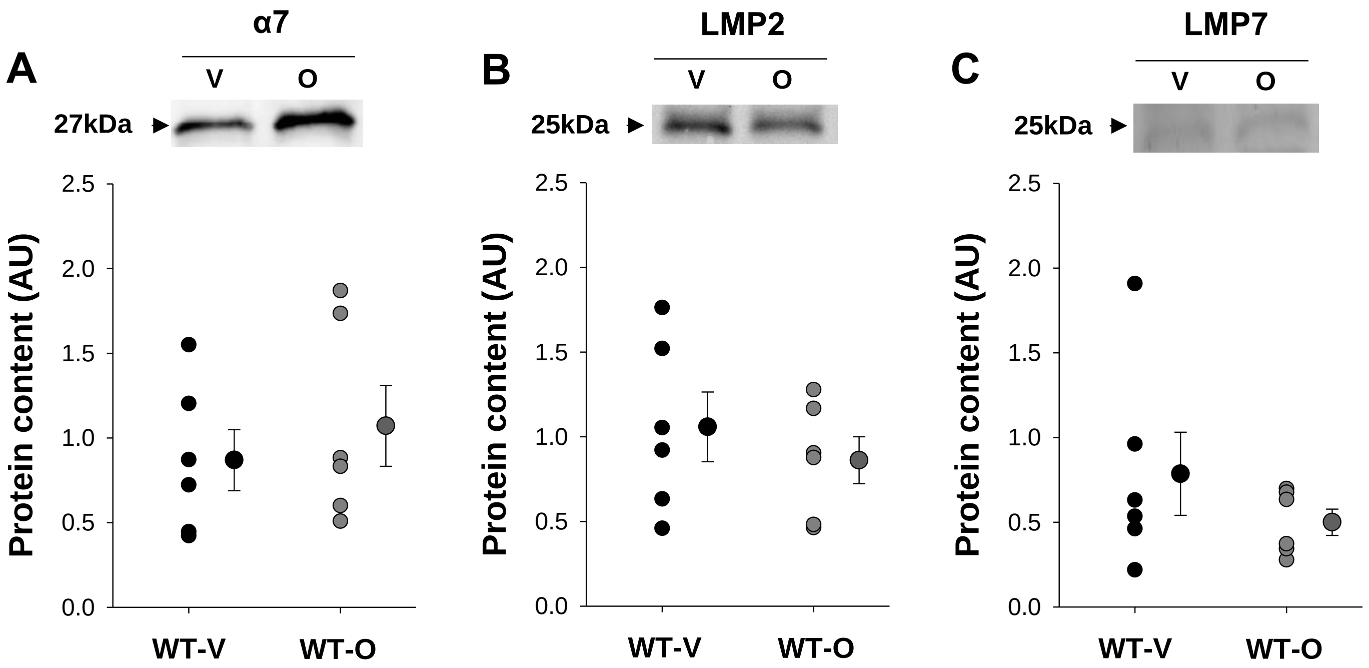

The content of α7, LMP2, and LMP7 in WT-V and WT-O mice. The content of α7(A), LMP2 (B), and LMP7 (C) of the gastrocnemius muscle in WT-V and WT-O mice was determined using Western blot analysis. Proteins were normalized to total protein and expressed as arbitrary unit (AU). Data presented as individual points (each mouse) with mean and standard error of the means (S.E.M.) * indicates $p<0.05$ comparing Vehicle to ONX-0914 treatment groups. Sample size: n=6 in each group.
